# Awareness and Knowledge of Glaucoma and Its Associated Risk Factors Among Adult Population in the Jazan Region, Saudi Arabia

**DOI:** 10.7759/cureus.48256

**Published:** 2023-11-04

**Authors:** Ismail Abuallut, Sahar Shubayli, Ghadeer Qumayri, Eman Refaei, Lojain I Daak, Mohammed Dibaji, Sarah M Salih

**Affiliations:** 1 Ophthalmology, Faculty of Medicine, Jazan University, Jazan, SAU; 2 Faculty of Medicine, Jazan University, Jazan, SAU; 3 Ophthalmology, Prince Mohammed bin Nasser Hospital, Jazan, SAU; 4 Community and Family Medicine, Faculty of Medicine, Jazan University, Jazan, SAU

**Keywords:** saudi arabia, jazan region, glaucoma, knowledge, awareness

## Abstract

Introduction

Glaucoma can be considered a heterogeneous group of diseases with characteristic progressive optic neuropathy, which causes the development of visual field dysfunctions and irreversible blindness. Glaucoma is the most prevalent cause of irreversible blindness worldwide.

Aim

This study aimed to assess the awareness and knowledge of glaucoma and its associated risk factors among the adult population in Jazan, Saudi Arabia, in 2022.

Methods

A community-based cross-sectional study was conducted with 384 participants aged 40 years and above in the Jazan region. A convenience sampling technique was adopted to obtain the target sample size. Data were collected using a self-administered electronic questionnaire on Google Forms.

Results

A majority (73.1%) of the 387 respondents to the questionnaire were in the age group of 40-50 years, with the average age being 47.43 years. No statistically significant difference was found between different age groups' level of knowledge (p=0.769). In addition, the difference between levels of knowledge based on participants' residence was also statistically insignificant (p=0.387). Of the participants who were not diagnosed with glaucoma, 55% had poor knowledge of glaucoma; however, only 3.10% of the participants diagnosed with glaucoma had poor knowledge of the condition. Consequently, there was a statistically significant difference between participants' levels of knowledge based on their diagnosis of glaucoma (p=0.04).

Conclusion

The study revealed low knowledge and awareness levels regarding glaucoma among adults aged 40 years and above in the Jazan region.

## Introduction

Glaucoma is a broad term for a set of diseases characterized by a gradual loss of retinal ganglion cells and the consequent loss of vision field [1]. It is the most prevalent cause of permanent blindness worldwide, affecting approximately 60 million individuals [2,3]. According to the World Health Organization, glaucoma is the second leading cause of blindness, after cataracts [4,5]. Its incidence is increasing as the world's population ages [1]. Glaucoma is frequently categorized into three major groups based on the genesis of the disease: open-angle, angle closure or closed-angle, and developmental. Open-angle glaucoma (OAG) and closed-angle glaucoma (CAG) are the two most common types of glaucoma. Primary angle-closure glaucoma (PACG) is the most common type of glaucoma in Asia, with recognized risk factors such as ethnicity, age, refractive error, sex, and family history. It is also the most common type of glaucoma in the United States, Europe, Africa, and Australia [6]. It is estimated that glaucoma, including OAG and ACG, will affect approximately 80 million people worldwide by 2020. Furthermore, approximately three-quarters of those affected will have OAG [7]. Glaucoma also affects 3.54% of people aged 40-80 years worldwide [8]. In a study conducted in Riyadh, Saudi Arabia, glaucoma awareness was shown to be lacking when compared to awareness of other eye disorders. The limited number of studies on chronic eye disorders in the Jazan region of Saudi Arabia also show that glaucoma is the least known disease in comparison with other diseases [9]. The most common glaucoma subcategories in Riyadh, Saudi Arabia, are primary open-angle glaucoma (POAG) (27.7%), secondary glaucoma (26.7%), PACG (18.2%), primary congenital glaucoma (2.7%), and juvenile OAG (2.2%) [10]. The epidemiology of visual impairment is linked to an increase in noncommunicable diseases, particularly diabetes, as well as other lifestyle-related factors such as dietary changes, sedentary lifestyles, and smoking [11]. Evidence on gender differences shows that menopause is a sex-specific risk factor: women with glaucoma have a higher risk of visual impairment and are also 24% less likely than men to seek glaucoma therapy [7].

A study in Jeddah Eye Hospital, Saudi Arabia, revealed low awareness and knowledge levels as well as misconceptions about risk factors, clinical features, and management of glaucoma among participants. Highly educated individuals and those with a positive history of diabetes or glaucoma scored high on knowledge [12]. According to several studies, glaucoma is the second leading cause of blindness after cataracts, and patients usually seek medical help at an advanced stage of the disease. This could be due to a lack of knowledge regarding glaucoma and its symptoms, as well as the natural progression of the disease marked by late appearance of symptoms [13]. Moreover, an obvious gap in the literature is present concerning the issue in the Jazan region specifically. Therefore, this study aims to address the issue of knowledge and awareness about glaucoma and its associated risk factors among the adult population in Jazan, Saudi Arabia. It is hoped that the evidence generated in this study will help improve awareness about the disease and the productivity and quality of life of patients.

## Materials and methods

This cross-sectional study was conducted in 2022 in the Jazan region, which is located in the southwest of Saudi Arabia and has a population of 1.6 million. This study includes all adults aged 40 years and above, living in the Jazan region, who completed the survey.

According to the General Authority for Statistics of the Kingdom of Saudi Arabia, there are 459,000 adults aged 40 and above in the Jazan region [14]. Accordingly, the sample size for this study was fixed at 384 participants and was calculated using the equation from rosoft.com. The parameters used in the study are as follows: p = 50% (to provide the maximum sample size), a 95% confidence interval, and a margin error of 5%.

The study sample was drawn from social media platforms from June to August 2022 using the convenience sampling technique until the target sample size was reached. Inclusion criterion was any adult 40 years old and above from the Jazan region with no other specific exclusion criterion. Data were collected using a self-administered electronic questionnaire designed by the researchers for this study. The questionnaire comprised three sections. The first section included six questions about age, gender, nationality, residency, education level, and history of glaucoma. The second section comprised three questions on awareness about glaucoma. The last section also covered glaucoma knowledge through 16 questions divided into four parts: risk factors, clinical features, types, and management of glaucoma. A consultant ophthalmologist assessed the face and content validity. Reliability testing of the knowledge scale based on the scoring system showed a Cronbach’s alpha of 0.88, indicating an acceptable internal consistency.

Data analysis software was used to review, code, and enter the extracted data. SPSS Statistics for Windows Version 22 (IBM Corp., Armonk, NY) was used. All variables, including demographic data and measures of awareness and knowledge, underwent a descriptive analysis based on the frequency and percentage distribution. Of the 19 questions on awareness and knowledge, 15 were surveyed. One point was scored for each correct answer. The scores obtained on different items were then summed up. Correct answers were assigned a value of 1, while incorrect answers were assigned a value of 0. This process was used to estimate a score indicating the knowledge level of each participant in the sample. Participants whose total score was less than 5 were considered to have poor knowledge. Those who scored in the range of 5-10 were considered to have intermediate knowledge, while participants who scored in the range of 11-15 were deemed to have good knowledge. Numerical data were assessed for normality using the Shapiro-Wilk test. Chi-square tests were used to correlate knowledge levels regarding glaucoma with demographic data. Statistical methods were verified, assuming a significance level of p < 0.05. The results of this study were then presented in tables and graphs.

A pilot study was conducted on 29 subjects to test the validity and accuracy of the questionnaire applied to respondents. In response to the results of the pilot study, certain questions were modified and rearranged. The final data analysis did not include data from the pilot study.

Ethical approval was obtained from the Research Ethics Committee of Jazan University, Saudi Arabia (Reference No. REC-43l/10/234) on May 22, 2022. Additionally, consent forms were obtained from all participants, and they were informed that the participation is totally voluntary and they can withdraw at any time.

## Results

A total of 387 adult participants in Jazan, Saudi Arabia, met the inclusion criteria and were included in the study. Their average age was 47.43 years, and the majority (73.1%) were in the age group of 40-50 years. The percentage of female participants was 63.3% and that of male participants was 36.7%. Almost all the participants were Saudi citizens. As regards residence, 55.6% lived in cities, while 44.4% lived in villages. Most participants (76.2%) had a university education. Only 6.5% of participants were diagnosed with glaucoma. Sociodemographic characteristics of the participants are presented in Table 1.

**Table 1:** Sociodemographic characteristics of the sample.

Factors		N=387	%
Age (years)	40-50	283	73.1
	51-60	84	21.7
	>60	20	5.2
Gender	Female	245	63.3
	Male	142	36.7
Nationality	Saudi	384	99.2
	Non-Saudi	3	0.8
Residency	City	215	55.6
	Village	172	44.4
Education	Illiterate	12	3.1
	Primary school	19	4.9
	Middle school	9	2.3
	Secondary school	52	13.4
	University	295	76.2
Diagnosis of glaucoma	Not diagnosed	362	6.5
	Diagnosed	25	93.5

**Figure 1:**
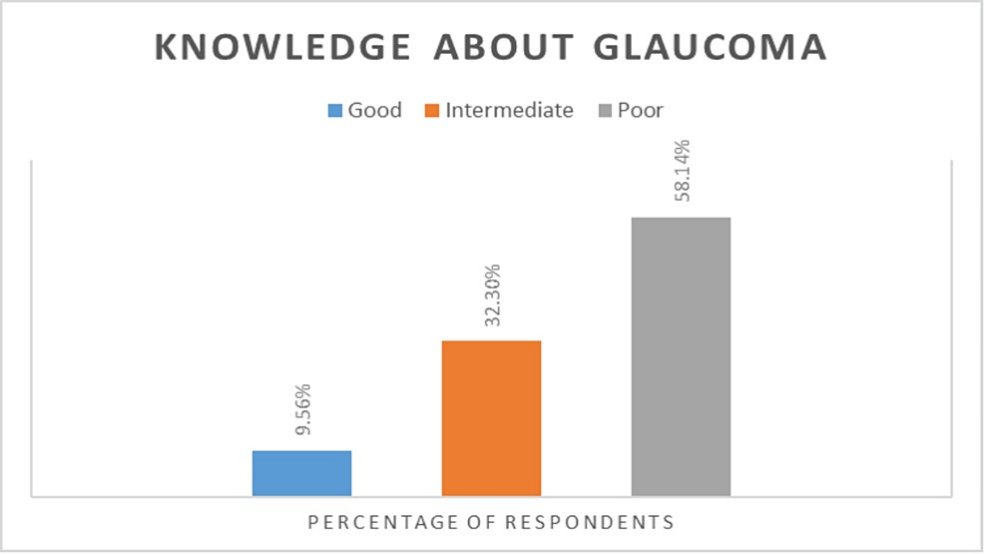
Distribution of knowledge score about glaucoma among the studied population

Most participants had a poor level of knowledge of glaucoma, as well as its types, symptoms, risk factors, and management (Table 2, Figure 1). Knowledge distribution by age indicates that most of the participants had poor knowledge, as observed among 59.4% of those in the age group of 40-50 years, 52.4% of those in the age group of 51-60 years, and 65% of those above the age of 60 years. There was no statistically significant difference between different age groups' level of knowledge (p= 0.769).

**Table 2:** Characteristics of the sample and the association with the knowledge level regarding glaucoma and its factors in the Jazan region.

Factors		Good	Intermediate	Poor	P-value
Age (years)	40-50	26 (9.2)	89 (31.4)	168 (59.4)	0.769
	51-60	9 (10.7)	31 (36.9)	44 (52.4)	
	>60	2 (10)	5 (25)	13 (65)	
Gender	Female	19 (7.8)	81 (33.1)	145 (59.2)	0.283
	Male	18 (12.7)	44 (31)	80 (56.3)	
Nationality	Saudi	37 (9.6)	125 (32.6)	222 (57.8)	0.337
	Non-Saudi	0 (0)	0 (0)	3 (100)	
Residency	City	19 (8.8)	72 (33.5)	124 (57.7)	0.387
	Village	18 (10.5)	53 (30.8)	101 (58.7)	
Education	Illiterate	2 (16.7)	1 (8.3)	9 (75)	0.387
	Primary school	0 (0)	4 (21.1)	15 (78.9)	
	Middle school	1 (11.1)	3 (33.3)	5 (55.6)	
	Secondary school	5 (9.6)	15 (28.8)	32 (61.5)	
	University	29 (9.8)	102 (34.6)	164 (55.6)	
Diagnosis of glaucoma	Not diagnosed	31 (8.6)	118 (32.6)	213 (58.8)	0.040
	Diagnosed	6 (24)	7 (28)	12 (48)	

**Figure 2:**
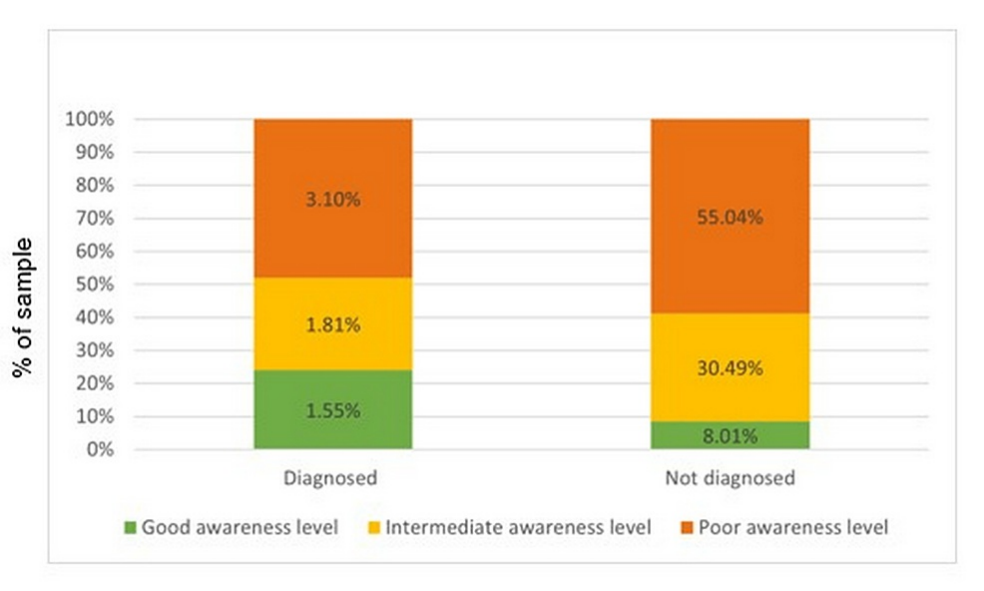
Association between the diagnosis of glaucoma and the knowledge level regarding glaucoma

The regional distribution of poor awareness levels was as follows. Around 58.7% of those residing in villages and 57.7% of those residing in cities had poor knowledge. Around 10.5% of the participants living in villages and 8.8% of those living in cities had good awareness. There was no statistically significant difference between participants' level of knowledge based on their area of residence (p = 0.387). The association between the diagnosis of glaucoma and the knowledge level of glaucoma is shown in Figure 2.

As regards the relationship between participants' level of education and knowledge, it was found that knowledge levels were weak at most levels of education. As shown in Table 2, 75% of the illiterate participants, 78.9% of those who completed primary school, 55.6% of those who completed middle school, 61.5% of those who completed secondary school, and 55.6% of those who completed university had poor levels of knowledge. There were no statistically significant differences between the levels of knowledge of participants and different education levels (p= 0.387).

Exploring the association between participants’ glaucoma diagnosis and their level of knowledge, good knowledge levels were reported in only 8.6% of participants who were not diagnosed with glaucoma, as opposed to 24% of the participants diagnosed with glaucoma. Intermediate levels of knowledge were reported in 32.6% of the participants who were not diagnosed with glaucoma and in 28% of those diagnosed with glaucoma. Finally, poor levels of knowledge were found among 58.8% of the participants who were not diagnosed with glaucoma and in 48% of those diagnosed with glaucoma. Therefore, there was a statistically significant difference between the participants' levels of knowledge based on their diagnosis of glaucoma (p= 0.04).

The results show that the prevalence of glaucoma in the studied population was 6.5% (n = 25). While 82.2% (n = 318) of participants had heard of glaucoma, 81.4% (n = 315) had previously undergone an eye examination by a doctor. With regard to participants' knowledge about the risk factors for glaucoma, 49.6% of the participants were aware that the risk of developing glaucoma increases with age, and 43.7% knew that people who suffer from high intraocular pressure are more likely to develop glaucoma. Similarly, 41% of the participants knew that diabetes and hypertension were risk factors for glaucoma. In addition, 19.9% of the participants recognized that previous eye injury was a risk factor for developing glaucoma. Furthermore, 15.0% of the participants recognized that genetic factors play a role in the risk of developing glaucoma and that a positive family history increases this risk.

Looking at participants’ knowledge of glaucoma’s presenting symptoms, 1.6% recognized eye redness, 5.7% recognized pain, and 21.2% recognized low visual acuity as presenting symptoms of glaucoma. Only 8.5% of the participants recognized all three - redness, pain, and low visual acuity - as presenting symptoms of glaucoma. Participants’ knowledge about the types of glaucoma showed that 8.53% of the participants knew about chronic OAG, 7.75% were aware of acute CAG, and 3.62% and 4.39% were aware of secondary glaucoma and congenital glaucoma, respectively.

As regards participants’ knowledge about the management of glaucoma, 55.8% knew it could be cured, and 20.9% were aware that glaucoma patients needed lifelong treatment. With respect to participants’ awareness of the different ways of managing glaucoma, 5.9% mentioned the use of eye drops, 10.9% pointed out eye surgery as an option, 2.6% stated laser, and only 24.5% recognized all three as options for glaucoma management.

## Discussion

This study assesses the level of awareness and knowledge regarding glaucoma among adults in the Jazan region of Saudi Arabia. A large number of participants had heard of glaucoma, but only 9.56% out of 387 had good knowledge about it (scoring 11-15 out of 15). While 32.30% had intermediate knowledge (scoring in the range of 5-10), 58.14% had poor knowledge levels (scoring below 5).

Our finding that 9.56% of participants had good knowledge about glaucoma is similar to that reported by Lau et al., whose results indicated that 10.2% of the total 2,538 participants had correct knowledge of glaucoma [15]. This may be due to the similarity in sociodemographic characteristics of the participants: in both studies, the participants were aged 40 years or above. However, the results of the present study indicate better participant knowledge in comparison with studies conducted in Uttar Pradesh, Nepal, and rural India, which reported that only 6.3%, 5.5%, and 1.89% had good knowledge about glaucoma, respectively [14,16,17]. This discrepancy could be attributed to differences in urbanization, access to health services, income, and level of education. Other studies conducted in Saudi Arabia reported that 16.9%, 18.8%, 24%, and 66.5% had good knowledge of glaucoma in Aljouf and Hail provinces, Jeddah, central Saudi Arabia, and Riyadh, respectively [12,18-20]. This difference in results does not necessarily mean that the population of the Jazan region is less aware and less knowledgeable about glaucoma; the different results could also be attributed to differences in sociodemographic characteristics, study design, setting, and statistical analysis of results. In the study by Al Zarea, the participants were aged 30 years and above [18]. Alqahtani et al. classified their participants into two groups, those with adequate and those with inadequate knowledge of glaucoma, based on a cutoff score [12], while in the present study, we classified participants into three groups: poor, intermediate, and good knowledge. The participants in the studies by Al Rashed et al. and Al Anazi et al. were those who presented to eye care clinics in Riyadh, Saudi Arabia [19,20]. Several studies have found that participants who visit an ophthalmology clinic at least once in their lives have greater knowledge of glaucoma [21-23]. This could be attributed to health education provided by ophthalmologists to patients undergoing eye examination. Therefore, direct comparison may not be applicable. These findings suggest the need for a standardized tool to assess knowledge and awareness of glaucoma.

We found no association of age or gender with glaucoma knowledge. These findings are similar to those of several previous studies [21,24-26]. However, some studies from developing countries have found a positive association between the male gender and knowledge of glaucoma [27,28], while other studies from developed countries have reported the opposite [29-31]. Unlike the present study, several studies have found an association between a particular age group and knowledge about glaucoma [21,27,31]. At the same time, there are several studies that have not found an association between age and knowledge [21,24-26]. In the present study, the difference in knowledge levels between rural and urban residents was not significant. This contrasts with other studies [13], which found that rural residents were lacking in knowledge about glaucoma in comparison with urban residents.

It is worth noting that several studies from around the world found an association between higher education levels and better knowledge about glaucoma [18,20,21,32-35]. This differs from our study, which suggests no association between education level and knowledge of glaucoma. The only demographic variable associated with knowledge of glaucoma in the present study was personal history of glaucoma. This is consistent with the findings of Alqahtani et al., who found that a personal history of glaucoma was significantly associated with better knowledge of glaucoma [12]. In contrast, a clinic-based study in the United States found that having a personal history of glaucoma was not associated with better knowledge of glaucoma [36]. Our results could be attributed to the health education provided to patients during their visit to the ophthalmology clinic. Alqahtani et al. found that 50.5% of participants obtained knowledge about glaucoma from physicians [12]. Therefore, physicians should consider their important role in health education. In addition, a diagnosis of glaucoma could encourage patients to search for more information. This suggests the need for reliable and easily accessible sources of glaucoma information.

The present study has a few limitations. First, sampling bias could be introduced by the use of convenience sampling technique. Second, response bias could arise due to the use of a self-reported questionnaire. These biases may undermine the generalizability of the results.

## Conclusions

This study reveals that only 9.56% of the participants had good knowledge about glaucoma and its associated risk factors. This highlights a significant gap in awareness and knowledge levels among the adult population in the Jazan region, Saudi Arabia. Our analysis found no statistically significant association between age or gender and glaucoma knowledge. This suggests that adult individuals and males and females have similar knowledge about glaucoma and its risk factors. Given the low awareness and knowledge levels observed in the study, it is imperative to implement comprehensive educational initiatives targeting the adult population in the Jazan region. We recommend developing targeted research that prioritizes high-risk groups, such as individuals with a history of glaucoma, older adults, and individuals with chronic conditions such as diabetes. These should provide tailored information and resources to address their needs and increase their understanding of glaucoma and its risk factors.
